# Nature-Based Guided Imagery as an Intervention for State Anxiety

**DOI:** 10.3389/fpsyg.2018.01858

**Published:** 2018-10-02

**Authors:** Jessica Nguyen, Eric Brymer

**Affiliations:** ^1^Department of Psychology, Queensland University of Technology, Brisbane, QLD, Australia; ^2^Institute of Sport, Physical Activity and Leisure, Leeds Beckett University, Leeds, United Kingdom

**Keywords:** anxiety, nature, guided imagery, anxiolytic, state-trait anxiety inventory

## Abstract

Anxiety is a significant mental health issue in modern society and empirical research into effective interventions to address anxiety has been extensive. Spending time in nature is one approach that has demonstrated anxiolytic effects. However, in some situations and contexts spending time in nature in order to reduce anxiety symptoms may not be possible. For example, in therapeutic settings delivered in a space with no access or exposure to any nature stimuli in the immediate surrounding environment. Guided imagery (GI) has also proven to be effective for reducing anxiety symptoms. Thus, nature-based GI might help to overcome the limitation of access to nature and strengthen the impact of GI interventions. The current study investigated the effectiveness of nature-based GI on anxiety reduction. Participants (*n* = 48, 18 males, 30 females, *M*_age_ = 34.54, *SD*_age_ = 12.91, age range = 19 – 71 years) with moderate levels of either trait or state anxiety as measured by the state-trait anxiety inventory (STAI) were recruited. Participants undertook both a nature-based GI session and a traditional non-nature-based GI session and their pre- and post- state anxiety levels were measured in each GI session. It was anticipated that post state anxiety scores would be significantly lower for both GI conditions and that a significantly greater anxiety reduction would be found in the nature-based GI than the urban-based GI. A two-way analysis of variance for repeated measures revealed results that supported both hypotheses. This study was the first to compare a nature based GI intervention with a traditional (non-nature based) GI intervention. Findings indicate that nature-based GI interventions are effective anxiety management interventions that have the added benefit of being cost-effective and easily accessible.

## Introduction

Anxiety is described as one of the world’s most debilitating mental health issues (World Health Organisation, 2011) and the development of effective interventions is fundamental to its successful management. In recent years there has been a considerable amount of research showing that nature benefits psychological health and wellbeing, including some research that indicates spending time in natural spaces is an effective intervention for the management of anxiety symptoms (Wheeler et al., 2012; Carrus et al., 2017; Fabjanski and Brymer, 2017; Lawton et al., 2017; Panno et al., 2017; Yeh et al., 2017; Schweitzer et al., 2018). However, a limitation of this approach is that contact with nature may not always be possible and some contexts and situations might make contact with nature more challenging. For example, living in high density urban environments or where therapy is undertaken in a confined room with no direct access to the natural world.

Guided imagery (GI) has been used as an effective intervention for anxiety by generating relaxing states through mental processes (Martin et al., 1999; Holmes and Mathews, 2005; Apóstolo and Kolcaba, 2009). An explicit addition of the natural environment to a GI process might serve to overcome the issue of physical access to nature and enhance the anxiolytic benefits of the GI process. Interventions using GI of nature might be an accessible and cost-effective intervention for anxiety reduction, as well as lend support for the growing evidence of the benefits of nature on mental well-being. This study is the first to investigate whether nature-based GI is an effective intervention for state anxiety. Findings have implications for the design and administration of effective anxiety interventions.

### Nature and Anxiety

Research indicates that nature can facilitate various positive psychological health and wellbeing outcomes (Wheeler et al., 2012; Carrus et al., 2017; Fabjanski and Brymer, 2017; Lawton et al., 2017; Panno et al., 2017; Yeh et al., 2017; Schweitzer et al., 2018). For example, experiences in nature have been shown to enhance vitality (Ryan et al., 2010), happiness (Capaldi et al., 2014), mood and self-esteem (Barton et al., 2011), and reduce stress (Kaplan, 1995). In recent years studies have found a link between nature and lower levels of anxiety and its antecedents (MIND, 2007; Mackay and Neill, 2010; Martyn and Brymer, 2014; Bratman et al., 2015a,b). For the most part research has focused on (1) the anxiolytic benefits of exercise in nature or (2) the relationship between feeling connected to nature and anxiety. Feeling connected to nature has been linked with lower overall anxiety. For example, a study conducted by Martyn and Brymer (2014) found that individuals with higher levels of connection to nature had significantly lower levels of overall state and trait cognitive anxiety (Martyn and Brymer, 2014). In particular, they found that physical familiarity with nature (experiential connection) was most significantly linked to lower anxiety. Lawton et al. (2017) compared levels of anxiety for regular indoor exercisers against regular outdoor exercisers and also found that physical familiarity with nature was most strongly linked to lower anxiety scores. They noted that exercising regularly outdoors predicted lower anxiety levels whereas exercising indoors predicted higher somatic anxiety. Both studies highlighted a correlation between an individual’s trait-based, subjective relationship with nature and low anxiety levels, thus providing evidence for a connection between nature and low anxiety. The study by Lawton et al. (2017) also suggested that spending time in nature augments the anxiolytic benefits of physical activity. However, neither study offered insights into nature’s capacity to act as a therapeutic intervention for anxiety.

Studies focusing on the relationship between physical activity in nature and anxiety have also found strong indicators for the anxiolytic benefits of nature. For example, a study commissioned by MIND (2007) reported that 71% of participants who walked in nature recorded less tension, whereas 50% of participants reported increased feelings of tension after a shopping center walk. Whilst this studies did not directly measure anxiety reduction, lowered stress and tension from engagement with nature alludes to nature’s potential anxiety reducing effects, as chronic levels of stress can result in anxiety (Vyas et al., 2004) and tension is a symptom of anxiety (American Psychiatric Association, 2013). Mackay and Neill (2010) found that exercising in green environments resulted in moderate short-term reductions in state anxiety. Greater anxiety reduction was experienced by those who perceived that they were exercising in more natural environments. Intensity and duration of physical activity did not impact state anxiety measures (Mackay and Neill, 2010). The authors suggested that ‘green’ environments are more likely to be restorative. Bratman et al. (2015a,b) argued that the anxiolytic effects of nature might come about because nature provides a rich set of the sensory stimuli that holds attention and reduces the harmful effects of rumination. Both Mackay and Neill (2010) and Bratman et al. (2015a,b) pointed out that research was needed to unpick the mechanisms by which nature experiences reduce anxiety.

While causal mechanisms are still unclear the above studies demonstrate a relation between nature and anxiety, and that physical exposure to the natural world may have the capacity to contribute to a decrease in anxiety states. However, as previously stated, direct contact with nature may not be possible and thus the question arises as to whether imagery of the natural world, through the process of GI, can lead to a reduction in anxiety.

### Guided Imagery and Anxiety

Guided imagery involves external instructional guidance to allow the internal generation of images (Hart, 2008), which invoke visual, auditory, haptic and taste-smell experiences as well triggering behavioral and physiological responses (Arbuthnott et al., 2001; Ji et al., 2016). GI can be utilized to encourage desired emotional and physical effects (Hart, 2008). Research has shown that under some circumstances, GI events are experienced as actual events (Kealy and Arbuthnott, 2003). This may be because the characteristics of the representations of GI events, such as the sensory and contextual detail, are similar to actual events. Further, there is evidence that visual mental imagery and visual perception share similar representations and are similarly processed (Borst and Kosslyn, 2008). Given the delivered and suggestive nature of GI, and its strong focus on contextual and sensory engagement, greater perceptual detail of the image is generated, creating a less discriminate difference between a real and imagined representation (Arbuthnott et al., 2002). Boschker et al. (2002) pointed out that in some instances while the processes involved in imagery and actual experiences are very similar the neuropsychological data suggests that imagery is not an exact representation of the real-world experience. Further, in these instances, imagery might actually be more effective than experiencing the real context because in imagery a participant might not focus on the unpleasant aspects of the context and instead focus on the most meaningful environmental characteristics. They use an ecological model to argue that the mutuality between person and environment is represented in imagery as the person actively imagines the realization of particular action possibilities. Interestingly, they assessed for demand characteristics in their study and found that while participants who were told that imagery did not work reported significantly lower capacity to imagine and reproduce an action, they were still able to reproduce the action as required. Boschker et al. (2002) concluded that while demand characteristics were influential, they were only partially important.

Numerous studies, across a wide range of populations, have demonstrated a link between GI and anxiety (Ayres and Hopf, 1985; Speck, 1990; Stephens, 1992; Rees, 1995; Casida and Lemanski, 2010; Thomas and Sethares, 2010; Vineeta et al., 2010; Serra et al., 2012). For example, a study conducted by Holmes and Mathews (2005) found that imagery of aversive events led to greater reporting of increased anxiety, as opposed to when the same aversive events were described verbally. The researchers concluded that imagery is especially powerful for anxiety symptoms because anxiety is a foundational ‘perceptually based emotion’ (p. 496) more likely to be responsive to perceptual-sensory representation than representational systems (e.g., language) that evolved later than these basic emotions. A review carried out by Holmes and Mathews (2010) concluded that imagery acts as an ‘emotional amplifier’ (p. 359) with the capacity to modify emotional states.

As an intervention for anxiety, there is a strong evidence base for GI in anxiety management (Ji et al., 2016) in a variety of contexts. For example with nursing students (Speck, 1990; Stephens, 1992), patients coping with medical-related anxiety (Casida and Lemanski, 2010; Thomas and Sethares, 2010; Vineeta et al., 2010; Serra et al., 2012), first time mothers (Rees, 1995), and individuals with speech anxiety (Ayres and Hopf, 1985). For example, Serra et al. (2012) conducted 30-min GI sessions with patients undertaking radiation treatment. The GI sessions started with systematic breath awareness, followed by visualization of a place where the patients felt most safe and comfortable. Examples of this place were given, including a favorite beach, park or any other location that patients found peaceful. However, it is not clear from the methodology as to whether these visualized places were verified to determine the specific content of the imagery experienced by the patients. Patients were then asked to concentrate on the sensory aspects associated with their image. The average number of GI sessions undertaken by participants in this study was between one and four, and anxiety measures were taken at the first session and the last session. The study found that participants reported a reduction in anxiety between the first session and the last session, with 86% of the participants describing the GI sessions as helpful (Serra et al., 2012). One study (Parnabas and Mahamood, 2012) that explored the relationship between visualizing imagery, nature and anxiety found that athletes who experienced higher levels of visualized green-space imagery experienced lower levels of competitive state anxiety. However, as many of the GI protocols in the above studies included phrases that actively encouraged participants to relax it is also possible that outcomes obtained were due to these instructions, making it difficult to ascertain whether anxiety reduction emerged from nature or the state of relaxation activated. Despite the potential links between imagery of nature and anxiety reduction, we were unable to find any studies that directly sought to investigate nature-based GI as an intervention for anxiety.

While these abovementioned studies did not explicitly and solely utilize imagery of the natural world within the GI process, they point to an intriguing possibility that GI using nature might provide augmented outcomes. In theory, the ecological explanation of how GI facilitates a multi-sensory focus on aspects of the imagined environment perceived to be most safe or comfortable, suggests that GI of both urban and natural environments have anxiolytic potential. From an ecological perspective GI using nature might be more effective because the natural world contains a richer multi-sensory landscape (Brymer et al., 2014; Yeh et al., 2016) and from an evolutionary perspective human perceptual systems are more likely attuned to information in the natural environment than the urban environment.

### The Current Study

Nature-based GI and its effectiveness in anxiety reduction is important to investigate as an intervention as it can serve to enhance the use of GI for anxiety reduction and overcome problems with regards to access to nature. The overall aim of the current study was to investigate whether nature-based GI reduces state anxiety. Two hypotheses were proposed. Hypothesis one posited that GI as a process itself reduces anxiety. Hypothesis two proposed that a nature-based GI would be more effective at reducing anxiety than a non-nature-based GI experience. As an individual’s relationship to nature or their ability to create mental images may influence their nature-based GI experience, measures of connectedness to nature, relatedness to nature and vividness of mental imagery were also obtained to assess any possible impact on the results.

## Materials and Methods

This study used a within-group design to compare a non-nature based GI intervention for anxiety with a nature-based GI intervention for anxiety. In order to compare the extent of anxiety reduction between the current study’s two interventions, a two-way repeated measures design ANOVA was employed. The within-group factors were the condition (nature vs. non-nature) and the time (pre vs. post). Order effects were checked via a counterbalance design of a two-way mixed design ANOVA. The between group factor was the order in which the participants undertook the condition; that is, participants were randomly assigned and either took the nature or non-nature condition first.

### Participants

A total of 48 participants completed the study in its entirety (18 males, 30 females, *M*_age_ = 34.54, *SD*_age_ = 12.91, age range = 19 – 71 years), 95.8% of participants resided in Australia and 4.2% resided in South-East Asia. Participants identified with a range of ethnic backgrounds (Caucasian = 67%; South-East Asian = 27%; Indigenous Australian = 2%; South European = 2%; Mixed = 2%). Eighteen participants took part in the first GI condition but not the second. Within the urban condition, 13 participants did not go on to complete the nature condition, 11 of these provided a baseline measure of anxiety. These 11 participants’ pre-intervention anxiety scores were compared to the remaining participants who completed the entire study. These participants did not differ significantly in their anxiety score and hence, their exclusion from the data set did not introduce undue bias, *t*(57) = 0.14, *p* = 0.889. Within the nature condition, five participants did not go on to complete the urban condition, with three of these providing a baseline measure of anxiety. Two of these participants scored the minimal possible score on the measure and therefore, possibly had less investment in continuing in an intervention designed to reduce anxiety, despite initially meeting screening requirements for a presence of anxiety symptoms.

To qualify for the study participants needed to be 18 years or over, and suffering anxiety symptoms. These requirements were assessed through an online screening questionnaire, which asked for the participant’s age and calculated the participant’s trait and state anxiety levels based on the State-Trait Anxiety Inventory (STAI) (Spielberger, 1983). The STAI manual (Spielberger et al., 1983) indicates that higher scores reflect higher anxiety, and suggests a cut-off score of 39 to differentiate between high and low anxiety. Normative data for the general Australian adult population suggest a normative STAI mean of 33 and 36 for state and trait anxiety, respectively (Crawford et al., 2011). The current study did not focus purely on clinical anxiety levels or a specific group, which carry varying cut-off points depending on the population. The current study set the cut-off score at 39, as this score suggests truly elevated levels of anxiety in individuals of the general Australian adult population. Any reduction from a score of 39 or more, would allow for significant changes in anxiety to be identified.

Participants were recruited online via a range of platforms and would have been aware of what the study entailed as the recruitment flyer was titled, ‘The role of nature in reducing anxiety through GI.’ Recruitment through social media platforms included posting the study details in Facebook pages and LinkedIn groups which could be identified as psychology or nature interest groups. Requests to external organizations to promote the study on their websites included private psychology practices and environmental organizations which showed interest in the mental health benefits of nature. A university study recruitment forum also advertised the study, offering first year psychology students course credit for completing the study. No monetary or other compensatory incentives were offered to participants. Data collection for the study was conducted anonymously and entirely online, with participants able to undertake participation at home, in their own time.

### Apparatus and Instruments

The study was conducted online using quantitative questionnaires and one qualitative question. The qualitative question was included to identify the type of environment imagined for each GI intervention in order to investigate compliance and identify themes. The question, ‘what images did you see in your mind?’ was worded as a broad open question to allow for a wide range of possible responses and to minimize the chance of leading responses.

#### Anxiety

The State-Trait Anxiety Inventory (STAI; Spielberger, 1983) is a 40 item self-report questionnaire that assesses state and trait anxiety levels. The 20 items assessing trait anxiety were used in the screening process to determine participant suitability to the study. These items require participants to report on a 4-point scale how frequently they experienced anxiety-related feelings and cognitions (1: Almost never; 4: Almost always). Example items include, ‘I am a steady person’ and ‘I lack self-confidence.’ The 20 items that measured anxiety as an emotional state were also used in the initial screening process for participant suitability. Items in this section required that participants respond to on a 4-point scale (1: Not at all; 4: Very much so) based on their feelings of anxiety ‘right now.’ Example items include, ‘I feel at ease’ and ‘I feel upset.’ The STAI was utilized for its brevity and excellent psychometric properties; construct validity is supported (Spielberger, 1983) and test–retest reliability has been found to be 0.97 for trait anxiety and 0.45 for state anxiety (Metzger, 1976).

#### Vividness of Imagery

Marks’ Vividness of Visual Imagery Questionnaire (VVIQ; Marks, 1973) is a self-report measure of vividness of mental, visual images. Participants are required to imagine 4 suggested scenes and then self-rate the vividness of their visual imagery on select details on a 5-point scale [1: Perfectly clear and as vivid as normal vision; 5: No image at all (only “knowing” that you are thinking of the object)]. Example items include asking participants to visualize a rising sun and rating their ability to vividly visualize the certain details, such as ‘The sky clears and surrounds the sun with blueness’ and ‘A rainbow appears.’ The VVIQ has demonstrated an internal consistency of 0.88, as measured by Cronbach’s alpha (McKelvie, 1995).

#### Connectedness to Nature

The Connectedness to Nature Scale (CNS; Mayer and Frantz, 2004) measures an individual’s trait levels of emotional connection to nature. The scale consists of 14 items and responses are rated on a 5-point Likert scale (1: strongly agree; 5: strongly disagree). Example items include, ‘I feel a kinship with animals and plants’ and ‘I often feel disconnected from nature.’ The CNS has been found to have good validity and to be reliable (α = 0.82) (Mayer and Frantz, 2004).

#### Relatedness to Nature

The Nature Relatedness Scale (NRS; Nisbet et al., 2009) is a 21-item scale that assesses the affective, cognitive and experiential aspects of individuals’ connection to nature. Responses are rated on a 5-point Likert scale (1: strongly disagree; 5: strongly agree). Example items include, ‘I am very aware of environmental issues’ and ‘I think a lot about the suffering of animals.’ The Nature Relatedness Scale has been found to have good validity and high reliability (α = 0.87) (Nisbet et al., 2009).

#### Guided Imagery Audios

Two GI audios were used as the intervention conditions. Both GIs were identical in process but differed in content; that is, one was the GI of a nature environment and the other was GI of an urban environment. The scripts were developed by the researchers in collaboration with a specially-trained psychologist who utilized mental GI in their professional practice. The process of script development firstly involved planning the script, by the steps outlined by Williams et al. (2013). This involved considering who will be using the script, the content of the imagery, the reasons for utilizing the script and consideration that the script will be delivered through an audio recording (Williams et al., 2013). As the recommendations by Williams et al. (2013) were intended for developing scripts for competitive athletes, the researchers used these steps as a guide and adjusted the athlete-related content to reflect the development of an urban- and nature-based GI script. General information on creating positive mental imagery (Stein, 2013), as well as the guidelines of the PETTLEP (Physical, Environment, Task, Timing, Learning, Emotion and Perspective) Model of Imagery (Holmes and Collins, 2001), were considered and the researchers studied a wide range of imagery scripts for further generation of ideas and to identify common and relevant elements. The GI scripts were then drafted based on the considerations and knowledge gained from the planning stage. The draft scripts were then reviewed by another psychology professional and feedback was incorporated to create the final, completed scripts. The final scripts were pilot tested on a volunteer, who did not recommend any further changes to the script.

The scripts focused on guiding participants to mentally engage with the environment through their senses. Neither script made any suggestions intended to actively invoke a state of relaxation, as the focus of the study was on the effect of the environment on anxiety, within a GI experience. Participants were asked, as part of the GI session, to take themselves to a place in nature or in an urban environment of their choosing, rather than being placed in a particular environment. This was important so as not to influence the environment in any way. However, in the urban-based GI session, participants were provided with examples of a possible urban environment. This was framed as follows: ‘Take yourself to a place in an urban environment of your choice. This may be a house you like, a new apartment building or a shopping mall, for example.’ The scripts were voice recorded and made into the form of a downloadable mp3 audio file, which participants could download and listen to from any listening device. No music or other sounds were included in the audio. The length of the GI audio was approximately 10 min.

### Intervention

The interventions in this study were the two GI sessions; one of which consisted of a nature-based environment, and another of an urban-based environment. Each participant undertook both conditions. Participants were randomly allocated to either the nature or the urban condition as the initial intervention: 23 undertook the nature-based condition first and the urban-based GI second, and 25 undertook the urban-based GI first and the nature-based GI second.

### Procedure

#### Screening and Information Gathering Phase

University ethical approval for human research was obtained prior to conducting the study. The entire study was conducted online. Successfully recruited participants completed a consent form which outlined eligibility requirements (i.e., age and current experience of anxiety), potential associated risks (such as exposure to natural/urban environments) and methods of management (contact details of psychological support services), and management of confidentiality (i.e., provided email addresses would be destroyed at the conclusion of the study). Once participants provided consent, participants generated an individual anonymous code which would be used to match participants to the results obtained from their future participation. The STAI was then administered and participants who scored ≥ 39 on either the trait or state anxiety scale qualified for the study. Demographic details and email addresses of the qualifying participants were then obtained. These participants then completed the VVIQ (Marks, 1973), the CNS (Mayer and Frantz, 2004) and the NRS (Nisbet et al., 2009). Participants were then emailed, at random, either the nature-based GI or urban-based GI audio, along with a set of emailed instructions, directing them to listen to the attached GI audio within the next week.

#### First Guided Imagery Session

Just before the participants undertook the first GI session, they were instructed to fill out a STAI questionnaire to assess their state anxiety. As mentioned above, the use of the STAI is appropriate for this study as it is an effective measure of state anxiety (Metzger, 1976; Spielberger, 1983) and its brevity allows it to be easily administered. Participants were then asked to undertake this GI session in a quiet environment where they would not be interrupted. Upon completion of the GI session, participants filled out another STAI questionnaire to measure their state anxiety after the GI session. Participants were also asked to provide key words to describe the content of the imagery generated in their minds. This information was obtained in order to verify that the imagery content related to the appropriate environmental category.

#### Second Guided Imagery Session

One week after the completion of the first GI experience, participants were sent the second GI audio file which contained the GI condition that they had not yet undertaken. The process for this second GI experience was the same as the first; i.e., pre- and post-state anxiety scores were obtained at the time the participant undertook the GI session and participants were asked to provide brief descriptions of their generated imagery content. Only participants who completed both GI sessions were included in the final analysis.

### Data Analysis

Quantitative data were prioritized over qualitative data (Hanson et al., 2005) and analyzed using the Statistical Package for the Social Sciences (SPSS). The quantitative data were checked for missing values. Little MCAR’s test returned an insignificant result, χ^2^ = 3.64, *p*(8) = 0.89 indicating that any missing value was completely random. Therefore, missing data was imputed using Estimation Maximization to create replacement values for missing data. A two-way mixed-design ANOVA was run to check for order effects, followed by the implementation of a two-way repeated measures ANOVA to determine the main analysis. Qualitative comments were thematically analyzed (Creswell, 2009). Comments were initially read to gather a sense of the overall experience. Topics were clustered by similarity and codes assigned. Descriptive categories were employed to reflect the aim of the study (Shaughnessy et al., 2009). Codes were cross checked by two researchers in an attempt to enhance reliability. Finally, quantitative and qualitative data were integrated to assist overall interpretation of results (Creswell, 2009).

## Results

The overall aim of the current study was to determine whether a nature-based GI experience was effective in reducing anxiety. The following analyses tested the hypotheses proposing that the process of GI itself reduces anxiety and that nature-based GI is more effective than non-nature-based GI at anxiety reduction.

The Shapiro–Wilk, F_max_ and Levene’s tests revealed that the assumptions for normality and homogeneity of variance were not violated. A two-way mixed-design ANOVA was employed and the impact of order effects were assessed. No interaction was found, Pillai’s trace = 0.007, *F*(1,46) = 0.32, *p* = 0.57, ${\text{η}}_{\text{p}}^{2}$ = 0.007. This finding indicates that the order in which each condition was undertaken did not impact the results. Therefore, the two orders were combined to examine the main analysis and to maximize power. Similarly, gender differences were explored with no significant gender by treatment effects found, *F*(1,46) = 0.005, *p* = 0.946. Hence, analysis results based on the total sample are presented rather than gender stratified results. Correlations between pre-post change scores for the Urban and Nature conditions and scores on the CNS, NRS and VVIQ were examined and minimal relationships were found (*r* ranging from.01 to.12). Therefore, in the interests of maximizing power, given the small sample size, these three variables were not used as covariates in the subsequent ANOVA analyses.

A two-way repeated measures ANOVA was undertaken and a significant interaction effect was found between the condition (nature vs. urban) and time (pre vs. post), Pillai’s trace = 0.101, *F*(1,47) = 5.29, *p* = 0.026, ${\text{η}}_{\text{p}}^{2}$ = 0.101. This indicates that the pre-post change in participants’ anxiety levels was significantly greater for those in the nature condition than those in the urban condition.

**FIGURE 1:**
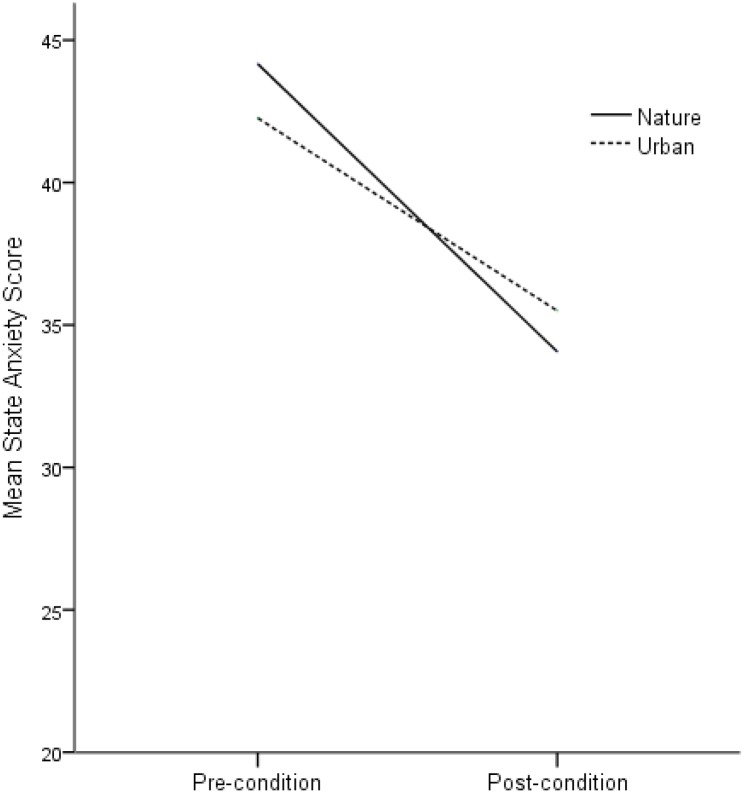
Pre to post changes in mean anxiety scores for nature and urban-based guided imagery intervention. Minimum score on state anxiety = 20.

The results reveal that both conditions were in themselves significantly effective in reducing anxiety. In the nature condition, the reduction in anxiety from before the participants undertook the condition to after the condition was significant, Pillai’s trace = 0.436, *F*(1,47) = 37.06, *p* < 0.001, *M*_diff_ = -10.10, 95% CI [-13.47, -6.73], ${\text{η}}_{\text{p}}^{2}$ = 0.436. In the urban condition, there was a significant reduction in anxiety from the pre-condition to the post-condition, Pillai’s trace = 0.342, *F*(1,47) = 24.40, *p* < 0.001, *M*_diff_ = -6.77, 95% CI [-9.52, -4.01], ${\text{η}}_{\text{p}}^{2}$ = 0.342. See **Table 1** for pre- and post-anxiety mean scores for both nature and urban conditions. See **Figure 1** for a graphical representation of the change in pre- and post-anxiety mean scores for both nature and urban conditions. **Tables 2**, **3** outline the themes and key phrases of the imagery generated in the nature-based GI and urban-based GI, respectively.

**Table 1:** Table of means for pre and post condition state anxiety scores.

	Pre		Post	
Condition	*M* (*SD*)	95% CI	*M* (*SD*)	95% CI
Nature (*n* = 48)	44.16 (11.90)	[40.71, 47.62]	34.06 (10.80)	[30.93, 37.20]
Urban (*n* = 48)	42.27 (11.39)	[38.96, 45.57]	35.50 (11.46)	[32.17, 38.83]

*CI, confidence interval*.

**Table 2:** Thematic category development of qualitative data from nature-based guided imagery.

Theme	Sub theme	Characteristic phrases
Water features	Beach	“Beach at the end of a cave,” “a beautiful beach I’ve been to before,” “beach environment – Fraser Island,” “looking out at the beach,” “on a beach sitting in the sand”
	Body of water (lake, river, ocean)	“River system leading to the ocean with reeds sprouting from the water,” “by a river”
	Waterfall	“Near waterfall,” “bottom of waterfall”
Mountain features	Mountains	“Blue mountains,” “green rolling mountains,” “mountains near childhood home,” “on top of a mountain”
Meadows and bush landscapes	Green area	“A grassy area near a river” “large green field surrounded by some hills,” “green meadow overlooking river and mountains,” “camping area…opposite green grassed area”
	Bush	“in Australian bush,” “where bush met trees”
	Nature landscapes/elements combination	“Forest and mountains in the background, and a field in the foreground which led to a clear blue lake”
Trees and forests	Forest/Rainforest	“rainforest trail,” “a pine forest with a creek,” “walking in a forest near a lake,” “forest with trees and bark,” “forest and lake covered in snow,” “tropical rainforest,” “rainforest track,” “forest next to grandmother’s farmhouse”
Backyard nature	Backyard	“In my backyard having an outdoor bath,” “backyard sitting in a recliner chair with my dog taking a nap beside me”

**Table 3:** Thematic category development of qualitative data from urban-based guided imagery.

Theme	Sub theme	Characteristic phrases
Home	Home	“Within my own home,” “current home,” “on my veranda of my previous home on acreage,” “childhood property with an ocean view and large leafy trees,” “small house,” “house that I used to live in,” “our townhouse,” “front door of my house,” “my townhouse complex”
	Apartment	“My apartment,” “new apartment building atrium area,” “outside an apartment building in a garden,” “rental apartment backyard”
Urban social	Café	“Alfresco café,” “busy café,” “an arcade of shops, cafes…I could smell coffee and all sorts of aromas coming from food”
	City	“City buildings,” “in the middle of the city shopping mall,” “city then forest,” “city, people, crowds, sidewalk…bustling, busy”
	Shopping center/mall area	“In the middle of the city shopping mall,” “Queen Street mall,” “outside a shopping center on a sunny warm day,” “shopping mall where I used to work,” “Chadstone shopping center in Melbourne, which is a busy area full of different shops and people walking around,” “an arcade of shops, cafés”
	Street	“Urban street scene beside a university campus,” “tree lined street”
Urban nature		“City then forest,” “tree lined street,” “in my backyard having an outdoor bath,” “colors of the room are white…there’s a window, in a wood frame, with the scenery outside is a lake, soft-blue sky,” “neighborhood, along a quiet road, near the forest,” “outside an apartment building in a garden,” “new apartment building atrium area”

## Discussion

The present study aimed to investigate the effect of a nature-based GI intervention on state anxiety reduction. Two hypotheses were proposed. Hypothesis one posited that GI reduces anxiety. Hypothesis two proposed that a nature-based GI would be more effective at reducing anxiety than a non-nature-based GI experience. Both hypotheses were upheld. Hypothesis one was supported as the results revealed that GI as a process reduced anxiety for participants in this study, as both the nature and urban GI conditions significantly reduced state anxiety. While it is always possible that just taking part in the study facilitated anxiety reduction, this finding aligns with the literature on GI and its ability to effectively reduce anxiety (Ayres and Hopf, 1985; Speck, 1990; Stephens, 1992; Rees, 1995; Casida and Lemanski, 2010; Thomas and Sethares, 2010; Vineeta et al., 2010; Serra et al., 2012). These results did not seem to be dependent on gender, imagery capacity or feelings of connection to nature. The mechanisms behind GI may provide further explanation of the results of the current study. One contributing factor for this reduction in anxiety may be due to the proposed ‘special link’ between imagery and anxiety whereby mental imagery seems to be especially useful for working with both the physiological and psychological symptoms of anxiety (Holmes and Mathews, 2005). Holmes and Mathews (2005, 2010) argued that foundational emotions such as anxiety were more readily manipulated though imagery, when compared to verbal representation, because imagery and the real experience both facilitate similar immediate perceptual experiences and directly influence similar emotional systems in the brain. Participants in this study were asked to evoke sensory experiences of the internally generated environment. For example, participants were asked to notice the smells and sounds in the environment that they had imagined. This focus on sensory detail by way of visual, auditory and tactile experiences allowed for greater perceptual detail of the generated image (Arbuthnott et al., 2001, 2002). This aligns with research that suggests that GI produces similar sensory responses as living the experience for real (Arbuthnott et al., 2002). Experiencing the ‘imagined’ environment as if it were real might facilitate similar reactions to being in the real environment. The ecological approach proposed by Boschker et al. (2002) suggests that the imagery process allows a participant to realize positive action possibilities within an ‘imagined’ environment. Participants are likely to have focused on the ‘best’ bits of the environment, ignoring the uncomfortable aspects. As participants knew that the study was intended to investigate anxiety reducing interventions participants might have actively imagined environments (urban and nature) that invoked anxiolytic experiences or safety and comfort (Serra et al., 2012). The current study did not assess the details of the GI experienced by the participants in depth, nor did it ascertain participants’ real-life responses to the particular environment they chose to experience. It may be possible that just taking part in the study facilitated anxiety reduction as participants knew they were taking part in a study examining the use of GI for anxiety. However, this still highlights the opportunity for further research into specific mechanisms underlying the reduction in anxiety brought about by both nature-based and urban-based GI experiences, such as the role of perception, sensory focus, memory associations and relationship to particular natural and urban environments.

Hypothesis two proposed that nature-based GI would be more effective at reducing state anxiety than the non-nature-based GI. The results supported this hypothesis. This reflects similar conclusions from previous studies comparing nature to urban conditions, which found that exposure to natural scenes triggered responses that may contribute to anxiety reduction, and does so to a greater extent than urban scenes (Ulrich, 1979, 1981; Parsons et al., 1998; Laumann et al., 2003; Bratman et al., 2015a,b). Participants were instructed to generate internal images of a natural environment of their own choice, and research has shown nature experiences can lead to lower heart rate, greater stress recovery and increase positive affect (Ulrich, 1979, 1981; Parsons et al., 1998; Laumann et al., 2003). It is possible that the action possibilities in the nature GI session provided a richer set of sensory-perceptual experiences that facilitated these positive responses and collectively contributed to the greater decrease in anxiety, perhaps facilitating reduced rumination (Bratman et al., 2015a,b). Interestingly, some participants included an aspect of urban nature within their urban imagery condition, though not vice versa (see **Table 3**). This is interesting for two reasons: first, though we feel the influence might be minimal, inclusion of nature in the urban condition might have contributed to the overall effects of the urban condition. Second, even though participants were explicitly asked to imagine urban areas, and given examples of urban scenes, participants still voluntarily brought aspects of nature into the experience. If, as noted above, participants invoked action possibilities that mirror safe and comfortable environments, it is interesting that, at least for some participants, even urban nature might provide imagined action possibilities that result in lower anxiety levels.

As the current study was the first study to investigate nature-based GI as an intervention for anxiety, it focused on the immediate effects of the guided-imagery experience on state anxiety. Future studies may want to explore the more long-term effects of nature-based GI interventions on anxiety levels by focusing on trait anxiety or the role of possible comorbidities, such as depression, on short and long-term outcomes. Further to this, future studies may also want to explore the effectiveness of nature-based GI on reducing anxiety for a clinical population, as participants in the current study displayed various levels of state anxiety. Additionally, the final participant pool for the current study mainly consisted of Australian participants, despite the study’s capacity to reach a more diverse population through its online recruitment process. These participants may have imagined aspects of the Australian landscape, which may be associated with more wide-open, natural landscapes. This is only a hypothesis as participants were only asked to provide a brief description of the GI environment using key words. Hence, future studies may be interested in exploring the effectiveness of nature-based GI interventions with participant groups from different geographical regions, such as landscapes that are more mountainous or more ‘urban-dense’ natural environments, and ensuring that participants’ GI environments adhere to specific environmental categories. Such research may help to highlight any possible differences between a range of natural environments on participants’ anxiety levels, as well as possibly providing greater clarification on the anxiety-reducing effect of different levels of greenery within urban environments. Future studies may also want to consider assessing the impact of the GI intervention with a non-contextual control group in order to further understand the imagery effects in different background contexts, as well as incorporating manipulation checks to ensure that participants experienced the GI exercise as anticipated.

The current study has demonstrated that nature-based GI is effective in reducing anxiety. It has also demonstrated that anxiety-reducing effects can emerge from imagery of the natural world itself without the need to incorporate suggestive relaxation cues, which is a common element in guided-imagery scripts. Future research could undertake a deeper exploration of the possible mechanisms underlying the reduction in anxiety seen in participants who experience nature-based GI and perhaps also investigate the efficacy of GI against similar interventions such as mindfulness training. Individuals with high trait mindfulness could be more responsive to GI interventions than individuals with low trait mindfulness because they are likely to pay more attention to presented stimuli. In this way, trait mindfulness might enhance the GI intervention. This could be achieved through comparative studies and qualitative interviews with the participants about their GI experiences.

Findings from this study have a number of implications. The most important implication of this study is that it highlights the many issues that require clarification in future studies. These directions for further research include disentangling the imagery effects of different locations and background contexts, differentiating the imagery effects of various levels of nature imagery and ascertaining the impact of previous experiences of the natural world on an individual’s anxiety-reduction. Practical implications from this study are fourfold. Firstly, imagery of nature can be appropriately incorporated into the design and administration of effective anxiety interventions whether they include nature or not. Secondly, health professionals can be confident in continuing to utilize the common intervention of guided-imagery, but better cater for nature-components of the exercise to suit the client’s personal affiliation to the natural world. This is because these findings have demonstrated that the content and features imagined by the individual during the guided-imagery experience is more important than the process itself. Thus, clinicians can seek to determine the relationship their clients have with particular natural environments and incorporate this knowledge into their GI-based interventions. Similarly, the knowledge that allowing individuals to create their own version of the environment, as opposed to situating them in a pre-determined environment, may be helpful in better aiding anxiety-reducing effects. Thirdly, nature-based GI can be undertaken anywhere as it can be experienced in the form of an audio recording, requiring only a listening device. This means that individuals can undertake nature-based GI outside of therapy, which in turn overcomes issues of access to therapy, such as waiting lists, limited therapy sessions or therapy costs, or barriers to therapy interventions that require direct contact with nature. Finally, the results of this study provide additional support for the growing body of evidence emerging in support of nature and its role in psychological well-being.

## Conclusion

Contact with nature has been shown to have anxiolytic effects. However, it may not always be possible to experience nature directly. GI has been used as an effective intervention for anxiety. This study set out to investigate the outcomes of using GI of nature as an intervention for anxiety. Results indicated that GI and nature-based GI were effective anxiety interventions. However, nature-based GI proved to be most effective. This finding has a number of practical implications and adds support to the growing evidence based that and enhanced human-nature relationship is important for psychological health and wellbeing. Evidence suggests that psychological interventions designed to develop positive experiences and boost positive behavior would be more effective if they enhanced the human-nature relationship in direct terms. However, if this is not possible then imagery of nature also has positive benefits.

## Ethics Statement

This study was carried out in accordance with the recommendations of Queensland University of Technology, research ethics. The protocol was approved by the Queensland University of Technology research ethics committee.

## Author Contributions

JN and EB contributed to design and analysis and article development. JN lead data gathering and analysis.

## Conflict of Interest Statement

The authors declare that the research was conducted in the absence of any commercial or financial relationships that could be construed as a potential conflict of interest.
